# Temporal trends in mortality related to infective endocarditis and stroke in the United States

**DOI:** 10.21542/gcsp.2026.10

**Published:** 2026-04-30

**Authors:** Sindhu P. Srinivasalu, Rekha Rao, Pranav Premkumar, Subhankar Panigrahi, Vaishnavi S. Joshi, Elizabeth S.R. Makka

**Affiliations:** 1 Apollo Hospitals. IIM, 154/11, Bannerghatta Rd, opposite Krishnaraju Layout, Krishnaraju Layout, Amalodbhavi Naga, Panduranga Nagar, Bengaluru, Karnataka 560076, India; 2 DC Clinic. No. 2, 3rd Cross Street, 3rd Main Road, Jaganathapuram, Velachery, Chennai, Tamil Nadu 600042, India; 3 Believers Church Medical College Hospital, St. Thomas Nagar, Kuttapuzha, Thiruvalla, Kerala 689103, India; 4 Bagchi Shri Sankara Cancer Care and Research Institute. Badaraghunathpur Road, Info Valley, Chandiheta, Bhubaneswar, Odisha 752054, India; 5 University of Galway. University Rd, Galway, Ireland; 6 Dr. Reddy”s Laboratories Limited. 8-2-337, Banjara Hills Rd Number 3, SBI Executive Enclave, Green Valley, Banjara Hills, Hyderabad, Telangana 500034, India

## Abstract

**Introduction**: Infective endocarditis (IE) remains a significant cause of mortality, and neurological complications—particularly stroke—are associated with worsened outcomes. However, temporal trends in mortality when both conditions co-occur remain underexplored.

**Aims:** To analyze mortality trends in infective endocarditis with stroke as a contributing cause of death using the CDC Multiple Cause of Death (MCD) database from 1999 to 2020.

**Methods**: A retrospective observational study was conducted using the CDC MCD database to assess mortality trends among individuals aged ≥25 years in the United States from 1999 to 2020. Deaths in which infective endocarditis (ICD-10: I33.0) was listed as the underlying cause and cerebral infarction (ICD-10: I63) as a contributing cause were analyzed. Data were stratified by age, sex, race, geographic region, and place of death. Age-adjusted mortality rates (AAMR) and annual percentage change (APC) were calculated.

**Results**: Between 1999 and 2020, 1,761 deaths met the inclusion criteria. The AAMR for IE with stroke demonstrated an initial decline followed by a subsequent increase (APC: 8.30%; 95% CI: 0.74–16.42). Mortality was highest among males, White adults, and those dying in metropolitan areas and medical facilities. Disparities were observed across demographic and geographic subgroups.

**Conclusions**:IE-associated stroke mortality shows a concerning upward trend, with significant disparities by sex, race, and location. These findings underscore the need for targeted prevention strategies and equitable healthcare access for high-risk populations.

## Introduction

Infective endocarditis (IE) is a life-threatening infection of the endocardial surface of the heart, most commonly involving the cardiac valves. Despite advances in antimicrobial therapy and diagnostic techniques, IE continues to carry substantial morbidity and mortality, with in-hospital mortality rates of 15–30%^1,2^. The epidemiology of IE is evolving, with increasing incidence among older adults, individuals with prosthetic valves, and those with cardiac implantable electronic devices^2,3^. In recent years, a marked rise in cases has been observed among people who inject drugs, particularly younger adults across both urban and rural communities^4,5^. Demographic disparities are well documented: males are disproportionately affected, and racial differences in outcomes persist, driven in part by delayed diagnosis and inequitable access to specialized care^6,7^.

Neurological complications, particularly stroke, are among the most severe and frequent manifestations of IE, occurring in approximately 20–40% of cases^8–10^. Ischemic stroke in this setting typically results from septic emboli originating from infected cardiac valves, leading to cerebral infarction^11^. Such events are associated with significantly increased in-hospital mortality and substantial long-term disability^9,12^. Moreover, the coexistence of IE and stroke complicates critical management decisions, including the timing of cardiac surgery, the role of anticoagulation, and the use of neuroimaging to guide treatment^13,14^.

The overlapping pathophysiology of IE and stroke—encompassing systemic inflammation, endothelial injury, and septic embolization—creates a clinically complex scenario with significant prognostic implications^9,11–14^. Although both conditions have been extensively studied in isolation, relatively few investigations have examined stroke as a contributing cause of death in the context of IE. Most prior mortality analyses have focused on IE as the primary cause of death, without accounting for the additional prognostic burden of coexisting neurological complications^15^.

The CDC Multiple Cause of Death (MCD) database offers a valuable opportunity to explore this intersection, capturing both underlying and contributing causes of death at a national scale. Leveraging this resource allows evaluation of temporal trends in IE-related mortality where stroke is recorded as a contributing factor, alongside an assessment of disparities across demographic and geographic subgroups^16^.

## Aims and objectives

To assess temporal trends in IE-related mortality where cerebral infarction is a contributing cause of death, using the CDC Multiple Cause of Death (MCD) database. This study analyzed mortality data from 1999 to 2020, stratified by sex, race, and geographic location, to identify disparities in mortality patterns across demographic and geographic subgroups.

## Methods

This retrospective observational study was conducted using the Centers for Disease Control and Prevention (CDC) Wide-Ranging Online Data for Epidemiologic Research (WONDER) Multiple Cause of Death (MCD) database^16^. The dataset comprises de-identified death certificate records from all 50 US states and the District of Columbia and is publicly available; accordingly, this study was exempt from institutional review board (IRB) approval^17–19^.

Data extraction was performed on January 20, 2025, and included deaths occurring between 1999 and 2020 among individuals aged ≥25 years. Infective endocarditis (ICD-10: I33.0) was designated as the underlying cause of death, and cerebral infarction (ICD-10: I63) as the contributing cause. Deaths not recorded with both conditions were excluded from the analysis.

Demographic variables included sex (male and female), race/ethnicity (White, Black or African American, Asian or Pacific Islander, and American Indian or Alaska Native), and geographic location classified by urbanization level according to the 2013 NCHS Urban-Rural Classification Scheme^20^. Metropolitan areas were categorized as Large Central Metro, Large Fringe Metro, Medium Metro, and Small Metro; non-metropolitan areas were categorized as Micropolitan and Non-Core. Place of death was recorded as medical facility, home, hospice facility, nursing home/long-term care facility, or other.

All mortality rates were age-adjusted per 1,000,000 population using the US Standard Population (year 2000) to ensure comparability across study years^21^. Descriptive statistics were used to report the number and percentage of deaths by demographic and geographic variables. Temporal trends in age-adjusted mortality rates (AAMR) were assessed using Joinpoint Regression Software (version 5.3.0.0, November 2024), which identifies statistically significant inflection points and calculates the annual percentage change (APC) with 95% confidence intervals (CI) for each trend segment^22^. Statistical significance was set at *p* < 0.05.

## Results

Between 1999 and 2020, the CDC Multiple Cause of Death (MCD) database recorded 1,761 deaths among individuals aged ≥25 years in the United States in which infective endocarditis (ICD-10: I33.0) was the underlying cause of death and cerebral infarction (ICD-10: I63) was recorded as a contributing cause. The crude mortality rate for IE with cerebral infarction as a contributing cause was 0.4 per 1,000,000 population. Deaths not meeting both criteria were excluded.

### Demographic characteristics

Of the 1,761 deaths analyzed, 965 (54.8%) occurred in males and 796 (45.2%) in females, indicating higher IE-related mortality with cerebral infarction as a contributing cause among males.

By race/ethnicity, the highest proportion of deaths occurred among White individuals (*n* = 1,445; 82.1%), followed by Black or African American individuals (*n* = 252; 14.3%), Asian or Pacific Islander individuals (*n* = 41; 2.3%), and American Indian or Alaska Native individuals (*n* = 23; 1.3%).

### Geographic characteristics

The majority of deaths occurred in metropolitan areas (*n* = 1,439; 81.7%), with non-metropolitan areas accounting for 322 deaths (18.3%). By place of death, most deaths occurred in medical facilities (*n* = 1,466; 83.5%), followed by hospice facilities (*n* = 126; 7.2%), decedents’ homes (*n* = 73; 4.2%), and nursing homes or long-term care facilities (*n* = 60; 4.3%). ‘Other place of death’ accounted for 0.8% of cases.

### Overall temporal trends

**Figure 1.**
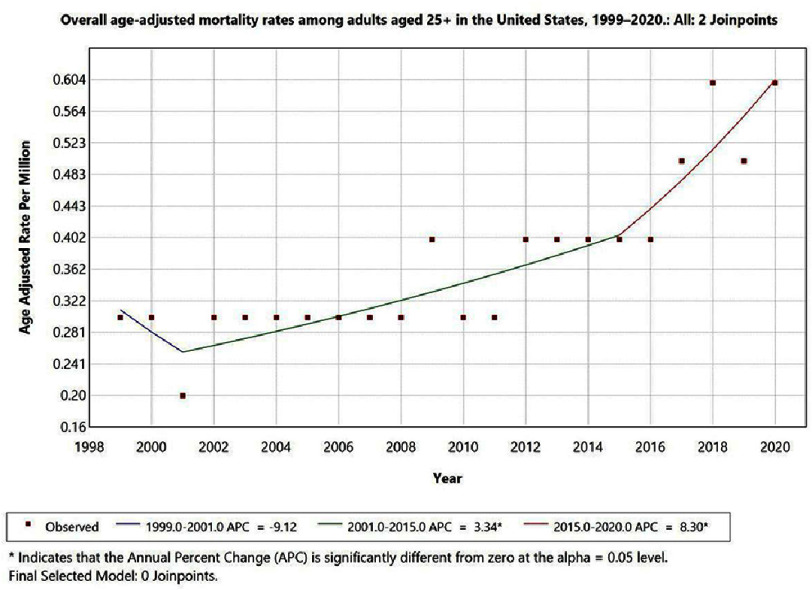
Overall age-adjusted mortality rates among adults aged 25+ in the United States, 1999–2020.

From 1999 to 2020, the AAMR for IE with cerebral infarction as a contributing cause demonstrated three distinct trend segments. An initial decline was observed from 1999 to 2001 (APC: −9.12%; 95% CI: −34.28 to 25.60; *p* < 0.05), followed by a period of gradual increase from 2001 to 2015 (APC: 3.34%; 95% CI: 1.60 to 5.10; *p* < 0.05). A steeper rise was subsequently observed from 2015 to 2020 (APC: 8.30%; 95% CI: 0.74 to 16.42; *p* < 0.05), suggesting an accelerating upward trend in recent years (Figure 1).

### Gender-stratified trends in age-adjusted mortality rates (AAMR)

When stratified by sex, distinct temporal patterns in AAMR were observed in males and females. Among females, three trend segments were identified over the study period. From 1999 to 2010, a modest increase in AAMR was observed (APC: 2.93%; 95% CI: −1.09 to 7.10; *p* < 0.05), suggesting a gradual rise in mortality burden. This was followed by a decline from 2010 to 2013 (APC: −8.82%; 95% CI: −49.49 to 64.59; *p* < 0.05). From 2013 to 2020, the AAMR increased sharply (APC: 12.19%; 95% CI: 3.67 to 21.40; *p* < 0.05), indicating a marked resurgence in mortality risk in the most recent period.

**Figure 2.**
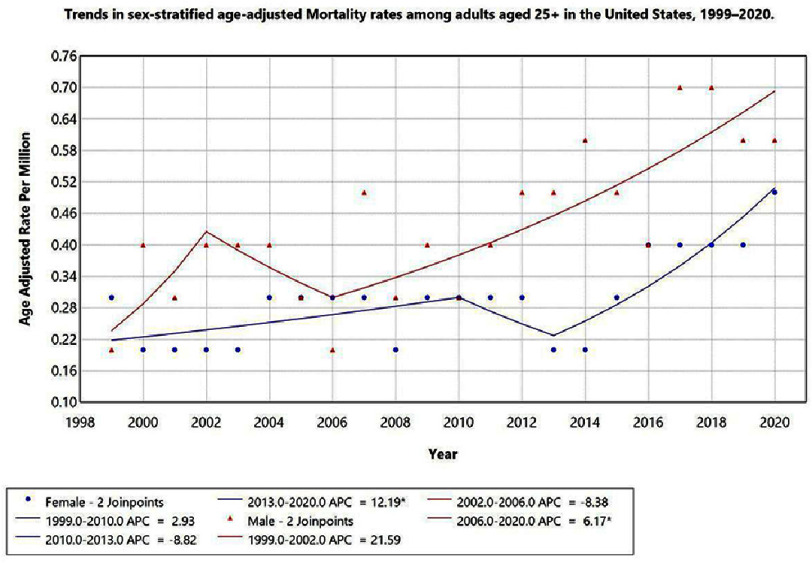
Trends in sex-stratified age-adjusted mortality rates among adults aged 25+ in the United States, 1999–2020.

Among males, three trend segments were similarly identified. A steep increase was observed from 1999 to 2002 (APC: 21.59%; 95% CI: −12.61 to 69.19; *p* < 0.05), followed by a decline from 2002 to 2006 (APC: −8.38%; 95% CI: −34.15 to 27.48; *p* < 0.05). From 2006 to 2020, the AAMR demonstrated a sustained upward trend (APC: 6.17%; 95% CI: 2.93 to 9.51; *p* < 0.05), representing a persistent increase in mortality risk over the longest trend segment observed. Both sexes demonstrated a resurgence in AAMR from approximately 2010 onward, with the most recent acceleration particularly pronounced in females (Figure 2).

### Race-specific trends

**Figure 3.**
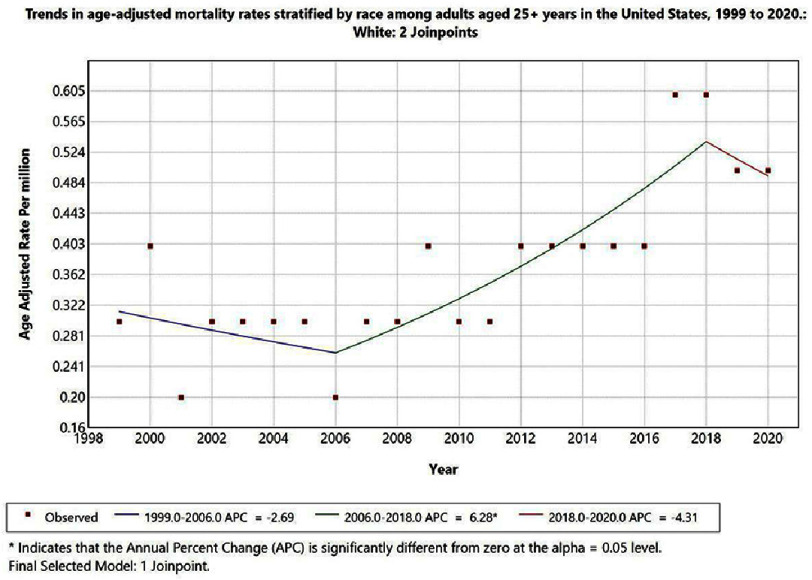
Trends in age-adjusted mortality rates stratified by race among adults aged 25+ years in the United States, 1999 to 2020.

Racial disparities were observed in IE-related mortality with cerebral infarction as a contributing cause. White adults had the highest AAMR over the study period, peaking at 0.58 per million in 2018. Three trend segments were identified for this group: a non-significant decline from 1999 to 2006 (APC: −2.69%; 95% CI: −9.25 to 4.35; *p* > 0.05), a significant and sustained increase from 2006 to 2018 (APC: 6.28%; 95% CI: 2.60 to 10.09; *p* < 0.05), and a subsequent non-significant decline from 2018 to 2020 (APC: −4.31%; 95% CI: −43.27 to 61.43; *p* > 0.05) (Figure 3). Reliable trend analysis for Black or African American, Asian or Pacific Islander, and American Indian or Alaska Native individuals could not be performed owing to data suppression for annual counts of fewer than 10 deaths.

## Discussion

This study examined national mortality trends in IE with cerebral infarction as a contributing cause of death among adults aged ≥25 years in the United States from 1999 to 2020. A total of 1,761 deaths met the inclusion criteria, with most occurring in medical facilities (83.5%) and predominantly among White individuals (82.1%) and males (54.8%). These findings are consistent with prior literature indicating that IE, although relatively uncommon, carries substantial mortality, particularly when complicated by neurological events such as stroke^1,2^.

Cerebral infarction is a well-recognized complication of IE, most commonly resulting from septic emboli originating from infected cardiac valves^7,8^. Approximately 20–40% of patients with IE develop embolic events, with the brain being the most frequently affected site^9^. Such neurological complications are associated with poorer prognosis and can complicate the timing and safety of surgical interventions—including valve replacement—that may be urgently required^10^.

The present analysis demonstrated an upward trend in IE-related mortality among both males and females over the study period. Among males, mortality increased significantly from 2006 onward, while among females a sharp rise was observed from 2013 onward. These trends may reflect multiple contributing factors, including an aging population, evolving patterns of healthcare utilization, and improved diagnostic practices leading to greater detection of severe cases^3,13^. Prior studies have also identified an association between the opioid epidemic and rising IE incidence, particularly among younger adults^4^; however, this relationship cannot be directly assessed using the present dataset.

Race-stratified analysis demonstrated that White individuals accounted for the majority of deaths in this cohort, which may reflect both underlying differences in disease burden and disparities in access to early diagnosis and specialized care. Data suppression for racial and ethnic groups with small annual counts precluded detailed trend analysis in minority populations, potentially leading to underrepresentation of mortality patterns in these groups. Prior studies have reported that racial and ethnic minorities may experience delays in diagnosis and reduced access to specialized cardiac and neurological care—disparities that may not be fully captured in population-level mortality datasets^5^.

Geographic analysis revealed that the majority of deaths occurred in metropolitan areas. This may partly reflect the greater concentration of advanced diagnostic facilities and specialist services in urban centers, facilitating detection and reporting of complex cases. Conversely, lower reported mortality in non-metropolitan areas may be attributable to underdiagnosis, delayed referral, or limited access to cardiac surgery, neurology, and critical care services—factors that may obscure the true burden of IE-related mortality in rural populations and contribute to disparities in outcomes^6^.

The high proportion of deaths occurring in medical facilities is consistent with the clinical severity of IE complicated by cerebral infarction, and aligns with prior reports demonstrating that such patients are more likely to require intensive care and experience prolonged hospitalization with high in-hospital mortality^14^.

From a public health perspective, the coexistence of IE and stroke represents a highly morbid and resource-intensive clinical condition. The present findings underscore the importance of early diagnosis and multidisciplinary management. Current American Heart Association guidelines recommend prompt initiation of antimicrobial therapy, timely surgical evaluation, and appropriate neuroimaging in patients with suspected embolic complications^1,10^. Disparities in access to these resources—particularly among underserved and non-metropolitan populations—likely contribute to the outcome differences observed in this study.

It is also important to note that 2020 (the final year of the study period) coincided with the COVID-19 pandemic, which may have influenced healthcare access, hospital utilization, and mortality reporting. Disruptions to routine medical care, delayed patient presentations, and strain on healthcare systems during this period may have affected both the diagnosis and management of IE and its complications. Accordingly, trends observed in 2020 should be interpreted with caution.

## Limitations

This study has several limitations. First, reliance on death certificate data introduces the potential for underreporting of contributing causes and inaccuracies in ICD-10 coding. Second, trend analyses for racial and ethnic subgroups were constrained by small annual counts and data suppression, limiting the generalizability of subgroup findings. Third, the MCD database lacks information on comorbidities, treatment history, socioeconomic status, and healthcare access - factors that may independently influence observed mortality patterns. Fourth, as a retrospective observational study, causality cannot be established; furthermore, changes in diagnostic criteria and coding practices over the study period may have introduced temporal inconsistencies in the data. Notwithstanding these limitations, the national scope of this analysis and its focus on the intersection of IE and cerebral infarction as a contributing cause of death represent meaningful strengths.

## Conclusions

IE-related mortality with cerebral infarction as a contributing cause demonstrated an accelerating upward trend over the study period, particularly among males and White individuals, with notable demographic and geographic disparities. These findings highlight the need for improved prevention strategies, early diagnosis, and timely multidisciplinary management. Efforts to reduce IE-related mortality must also address disparities in healthcare access and ensure equitable delivery of specialized cardiac and neurological care to high-risk and underserved populations.
